# 10a-Hy­droxy-9-[2-(tri­fluoro­meth­yl)phen­yl]-3,4,5,6,7,8a,9,10a-octa­hydro-1*H*-xanthene-1,8(2*H*)-dione

**DOI:** 10.1107/S2414314626008370

**Published:** 2026-08-20

**Authors:** Md. Mominuddin, Harendra N. Roy, Ennio Zangrando, Injamamul Hasan, Pran Gopal Karmaker

**Affiliations:** a Department of Chemistry, Rajshahi University, Rajshahi-6205, Bangladesh; b Department of Chemical and Pharmaceutical Science, University of Trieste, Italy; c Chemical Synthesis and Pollution Control Key Laboratory of Sichuan Provinces College of Chemistry and Chemical Engineering China West Normal University,Nanchong 637002 People’s Republic of China; Purdue University, USA

**Keywords:** crystal structure, xanthene, tetra­hydro­pyran, tri­fluoro­methyl­benzene

## Abstract

The title compound, C_20_H_19_F_3_O_4_, crystallizes with two independent mol­ecules with similar geometry. In both mol­ecules the tetra­hydro­pyran, cyclo­hexene and cyclo­hexane rings of the xanthene moiety adopt half-chair, half-boat and chair conformations, respectively. In the extended structure, O—H⋯O hydrogen bonds between hy­droxy and carbonyl groups link mol­ecules into chains propagating along [100].

## Abstract 2

The title compound, C_20_H_19_F_3_O_4_, crystallizes with two independent mol­ecules with similar geometry. In both mol­ecules the tetra­hydro­pyran, cyclo­hexene and cyclo­hexane rings of the xanthene moiety adopt half-chair, half-boat and chair conformations, respectively.

## Graphical abstract

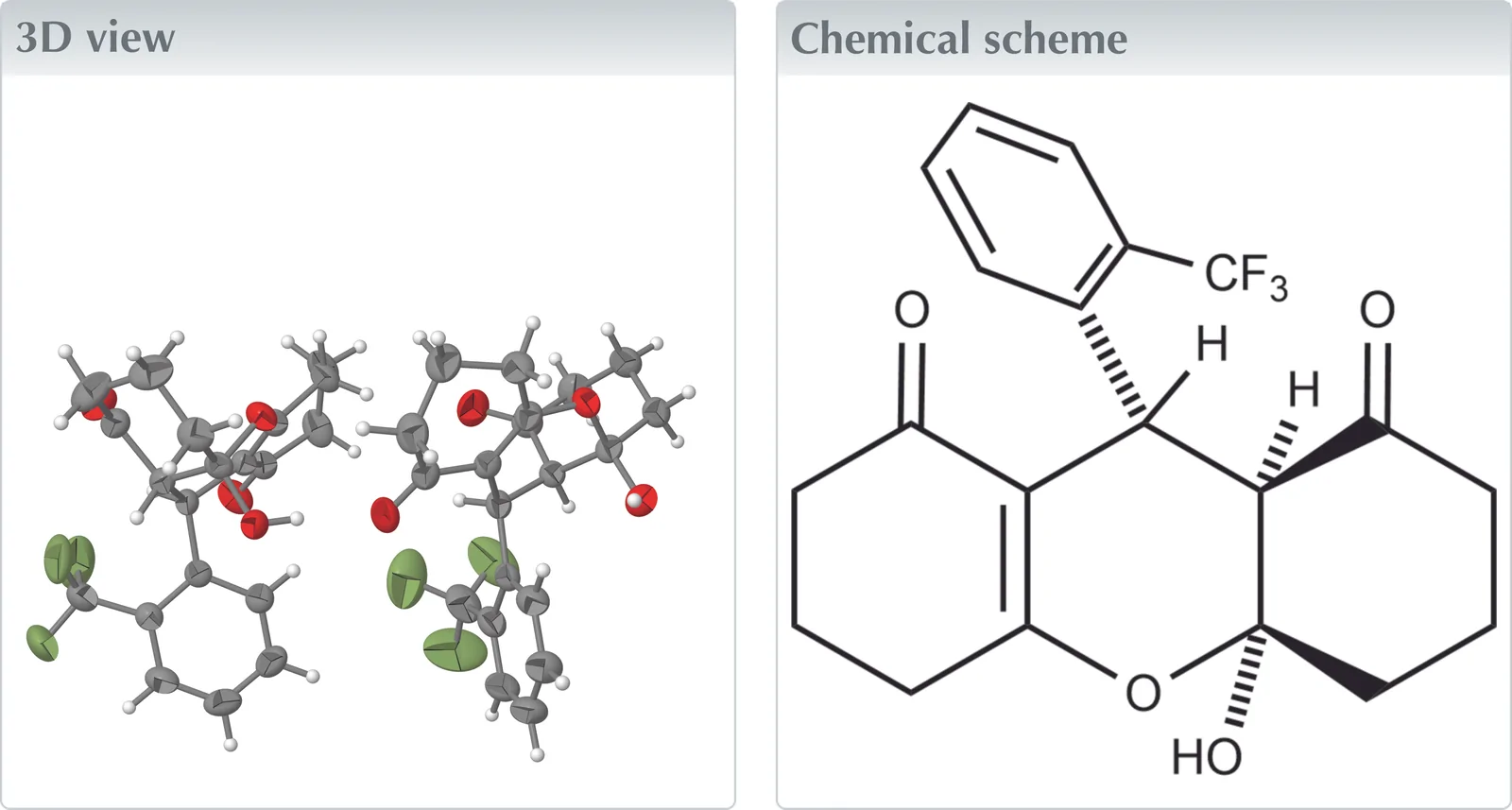

## Structure description

Xanthene derivatives constitute a prominent class of oxygen-containing heterocycles that are gaining attention owing to their biological activities and diverse industrial applications (Ran *et al.*, 2025 ▸, Taghartapeh *et al.*, 2017 ▸, Faryabi *et al.*, 2025 ▸).

**Figure 1:**
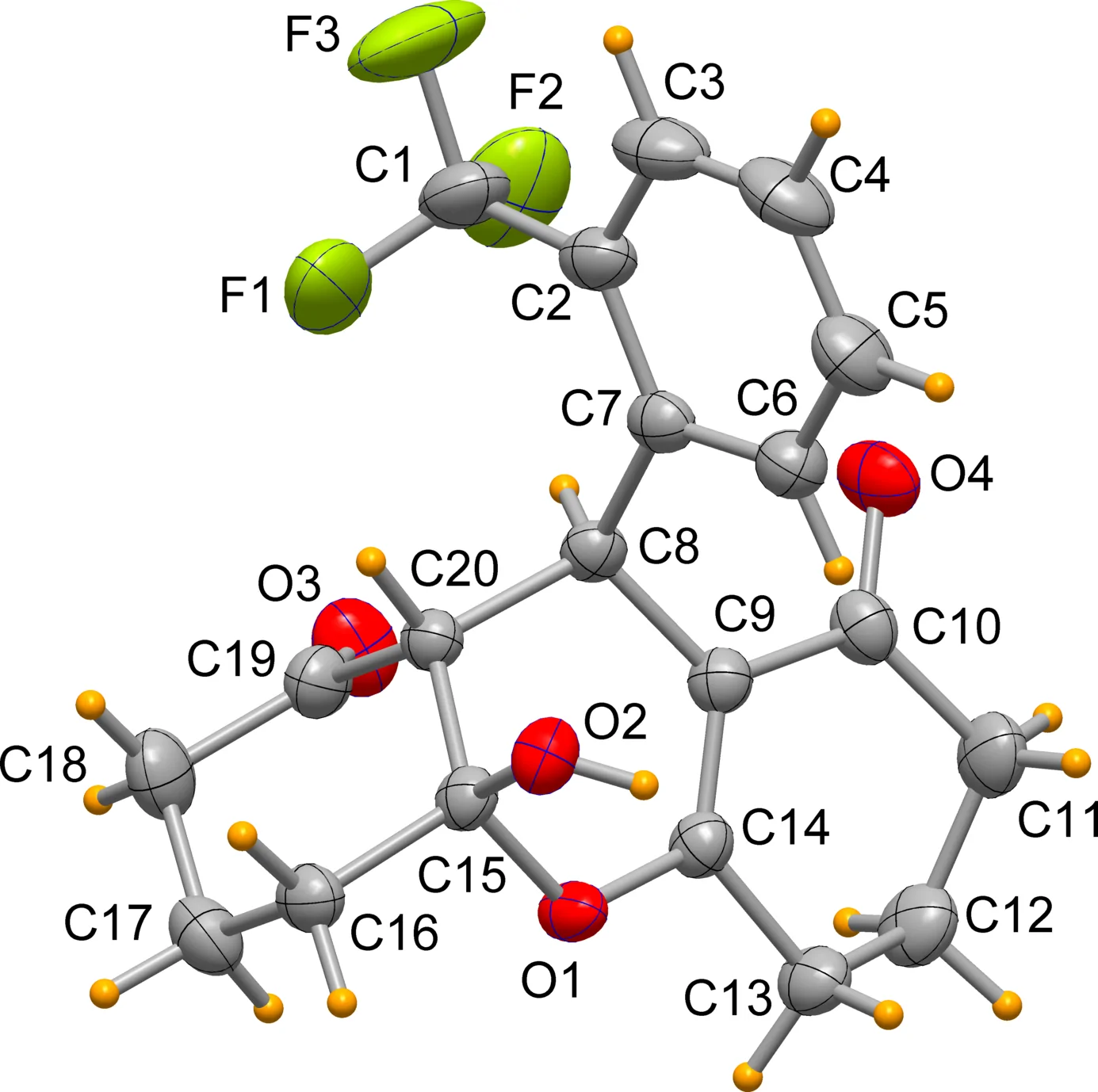
Displacement ellipsoid view (40% probability level) of mol­ecule 1.

The title compound, C_20_H_19_F_3_O_4_, crystallizes with two independent mol­ecules (1 and 2, shown in Figs. 1 ▸ and 2, respectively ▸), with similar geometry. The tetra­hydro­pyran rings adopts a half chair conformation with puckering parameters for mol­ecules 1 and 2 of *Q* = 0.448 (3) Å, θ = 126.4 (4)°, φ = 87.7 (5)° and *Q* = 0.451 (3) Å, θ = 54.5 (4)° φ = 268.9 (4)°, respectively. The cyclo­hexene (C9–C14 and C29–C34) and cyclo­hexane (C15–C20 and C35–C40) rings adopt half-boat and chair conformations, respectively. The mean plane of the tetra­hydro­pyran ring [r.m.s deviation = 0.183 and 0.184 Å] forms dihedral angles of 81.44 (9) and 78.75 (9) ° with the tri­fluoro­methyl­phenyl ring in mol­ecules 1 and 2, respectively. The crystal structure is further consolidated by intra­molecular C20—H⋯F1 and C40—H⋯F5 inter­actions (Table 1 ▸).

For related structures, see Ahmed *et al.*, 2024 ▸, Fun *et al.*, 2012 ▸, Karimian *et al.*, 2025 ▸, Li 2017 ▸, Li *et al.*, 2017 ▸, Patil *et al.*, 2025 ▸, and Pesyan *et al.*, 2020 ▸).

**Figure 3:**
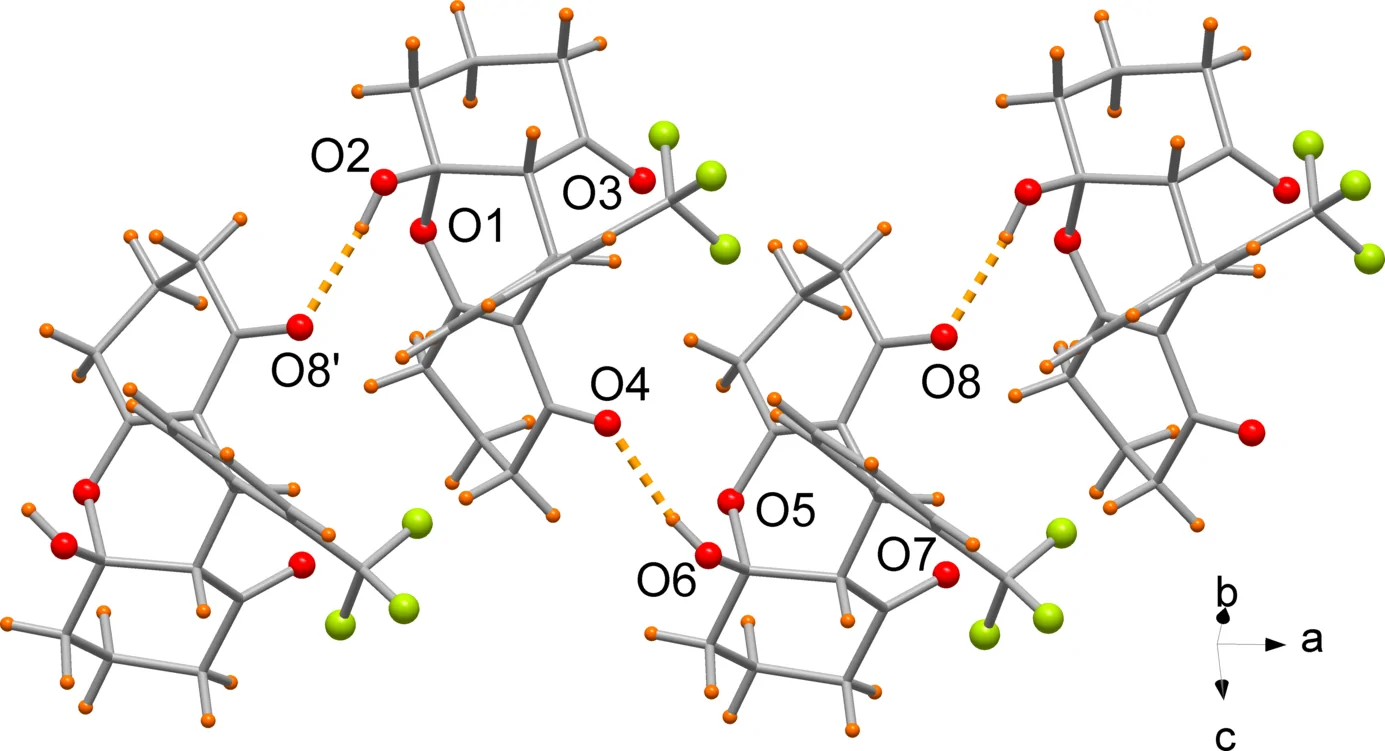
Crystal packing showing the mol­ecules hydrogen-bonded along the *a*-axis direction. Symmetry code: (′) *x* − 1, *y*, *z*.

In the crystal, the mol­ecules are linked by O2—H2⋯O8^i^ and O6—H6*A*⋯O4 hydrogen bonds (Table 1 ▸, Fig. 3 ▸), forming chains of alternating *A* and *B* molecules along [100]. Additional C—H⋯O inter­actions reinforce the structure.

## Synthesis and crystallization

1,3-Cyclo­hexa­ndione (2 mol), 2-(tri­fluoro­meth­yl)benzaldehyde (1 mol) and nicotinic acid (10 mol %) were placed into a round-bottom flask. All the starting components were initially mixed with a glass rod. Ethanol (5 ml) was then added to the flask and the reaction mixture was heated at 60–65°C over a period of 5 h with an efficient CaCl_2_ guard tube at the top of the condenser. After completion (TLC checked) single crystals were obtained by slow evaporation of the solvent.

The isolated yields was 90%. Color: white, m.p. 268–269°C; IR (KBr): (*v*) = 2945, 2922, 2866, 1650, 1569, 1492, 1359, 1181 cm^−1^.

**^1^H NMR (400 MHz, CDCl_3_):** δ (p.p.m.) = 12.3 (*s*, 1H, *t*-OH), 7.56–7.51 (*m*, 3H, Ar—H), 7. 45–7.39 (*m*, 1H, Ar—H), 5.72 (*s*, 1H, condensing carbon, *ali*-CH), 2.27–2.08 (*m*, 3H, *ali*-CH_2_), 2.07–1.81 (*m*, 5H *ali*-CH_2_), 1.80–21.72 (*m*, 5H, *ali*-CH_2_);

**^13^C NMR (400 MHz, CDCl_3_):** δ (p.p.m.) = 196.0 (C=O), 151.6, 139.2, 133.2, 130.9, 129.9, 129.4, 126.2, 115.8, 136.6, 33.8, 28.6, 21.1, 19.5;

**HRMS (ESI):** Calculated for C_20_H_19_F_3_O_4_: 380.1257, found [*m*/*z*, *M* + H]^+^ 381.1259.

## Refinement

Crystal data, data collection and structure refinement details are summarized in Table 2 ▸.

## Supplementary Material

Crystal structure: contains datablock(s) I. DOI: 10.1107/S2414314626008370/zl4102sup1.cif

Structure factors: contains datablock(s) I. DOI: 10.1107/S2414314626008370/zl4102Isup2.hkl

Supporting information file. DOI: 10.1107/S2414314626008370/zl4102Isup3.cml

CCDC reference: 2580767

Additional supporting information:  crystallographic information; 3D view; checkCIF report

## Figures and Tables

**Figure 2:**
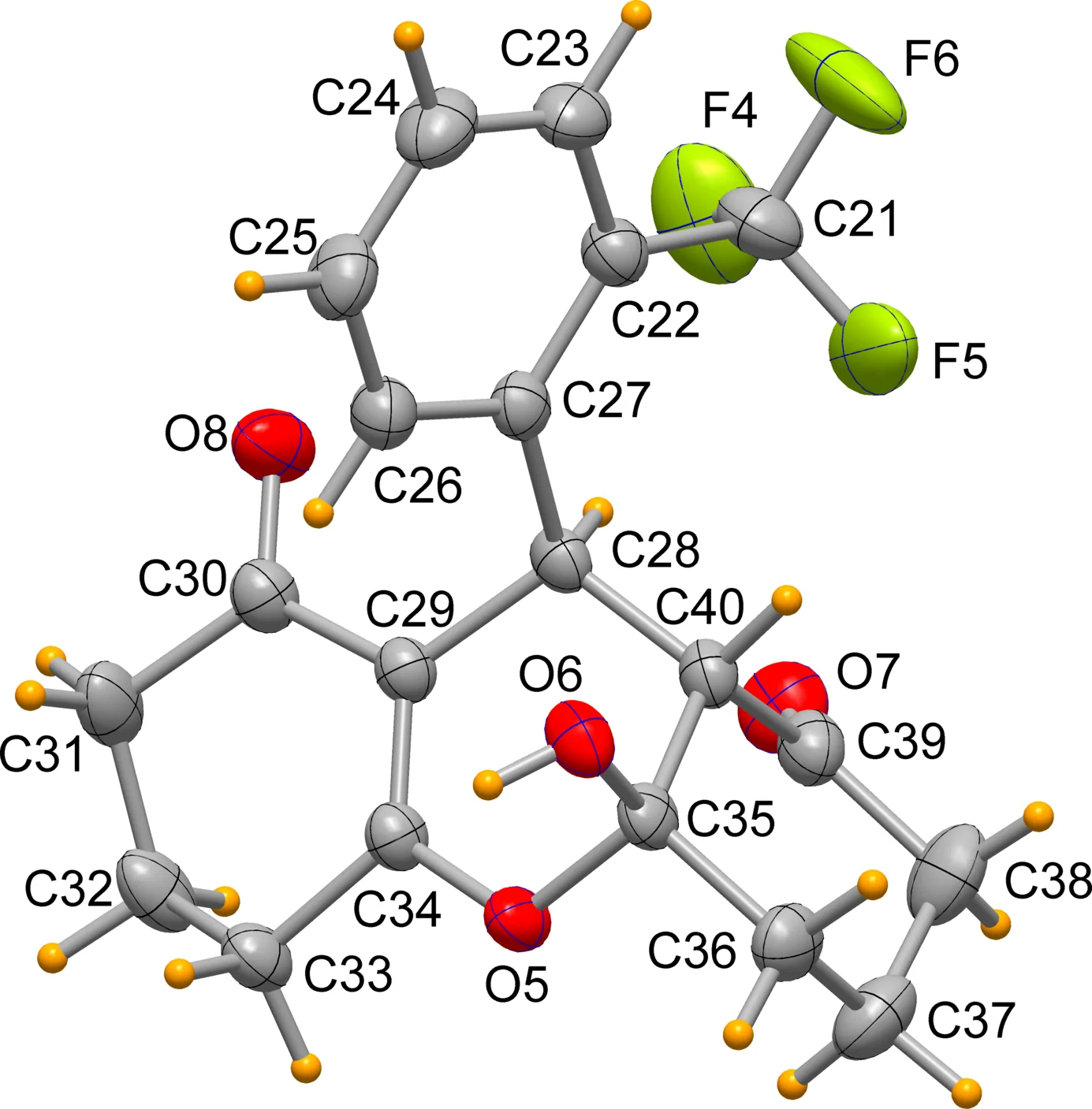
Displacement ellipsoid view (40% probability level) of mol­ecule 2.

**Table 1:** Hydrogen-bond geometry (Å, °)

*D*—H⋯*A*	*D*—H	H⋯*A*	*D*⋯*A*	*D*—H⋯*A*
C20—H20⋯F1	0.98	2.40	2.960 (4)	116
C40—H40⋯F5	0.98	2.35	2.901 (4)	115
C11—H11*A*⋯F4^i^	0.97	2.51	3.307 (5)	140
O2—H2⋯O8^i^	0.89 (4)	1.82 (4)	2.704 (3)	172 (3)
O6—H6*A*⋯O4	0.86 (4)	1.83 (4)	2.684 (3)	171 (3)
C16—H16*B*⋯O2^ii^	0.97	2.52	3.403 (4)	152
C36—H36*B*⋯O6^iii^	0.97	2.59	3.473 (4)	152

Symmetry codes: (i) 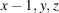; (ii) 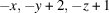; (iii) 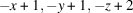.

**Table 2:** Experimental details

Crystal data	
Chemical formula	C_20_H_19_F_3_O_4_
*M*_r_	380.35
Crystal system, space group	Triclinic, *P* 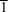
Temperature (K)	293
*a*, *b*, *c* (Å)	10.408 (3), 11.227 (4), 15.455 (5)
α, β, γ (°)	86.17 (1), 85.465 (9), 85.919 (10)
*V* (Å^3^)	1792.3 (10)
*Z*	4
Radiation type	Mo *K*α
μ (mm^−1^)	0.12
Crystal size (mm)	0.28 × 0.25 × 0.24

Data collection	
Diffractometer	Bruker APEXII CCD
Absorption correction	Multi-scan (*SADABS*; Krause *et al.*, 2015 ▸)
*T*_min_, *T*_max_	0.890, 1.000
No. of measured, independent and observed [*I* > 2σ(*I*)] reflections	47730, 6616, 4728
*R*_int_	0.064
(sin θ/λ)_max_ (Å^−1^)	0.605

Refinement	
*R*[*F*^2^ > 2σ(*F*^2^)], *wR*(*F*^2^), *S*	0.068, 0.150, 1.08
No. of reflections	6616
No. of parameters	493
H-atom treatment	H atoms treated by a mixture of independent and constrained refinement
Δρ_max_, Δρ_min_ (e Å^−3^)	0.49, −0.34

Computer programs: *SMART* and *SAINT* (Bruker, 2012 ▸), *SHELXT2014/5* (Sheldrick, 2015*a* ▸), *SHELXL2019/2* (Sheldrick, 2015*b* ▸), *DIAMOND* (Brandenburg, 1999 ▸) and *WinGX* (Farrugia, 2012 ▸).

## full crystallographic data

### 10a-Hydroxy-9-[2-(trifluoromethyl)phenyl]-3,4,5,6,7,8a,9,10a-octahydro-1H-xanthene-1,8(2H)-dione Crystal data

**Table:**

C_20_H_19_F_3_O_4_	*Z* = 4
*M**_r_* = 380.35	*F*(000) = 792
Triclinic, *P*1	*D*_x_ = 1.410 Mg m^−^^3^
*a* = 10.408 (3) Å	Mo *K*α radiation, λ = 0.71073 Å
*b* = 11.227 (4) Å	Cell parameters from 9858 reflections
*c* = 15.455 (5) Å	θ = 2.3–23.5°
α = 86.17 (1)°	µ = 0.12 mm^−^^1^
β = 85.465 (9)°	*T* = 293 K
γ = 85.919 (10)°	Block, colourless
*V* = 1792.3 (10) Å^3^	0.28 × 0.25 × 0.24 mm

### 10a-Hydroxy-9-[2-(trifluoromethyl)phenyl]-3,4,5,6,7,8a,9,10a-octahydro-1H-xanthene-1,8(2H)-dione Data collection

**Table:**

Bruker APEX-II CCD diffractometer	4728 reflections with *I* > 2σ(*I*)
φ and ω scans	*R*_int_ = 0.064
Absorption correction: multi-scan SADABS-2016/2 (Bruker, 2016) was used for absorption correction.	θ_max_ = 25.5°, θ_min_ = 2.3°
*T*_min_ = 0.890, *T*_max_ = 1.000	*h* = −12→12
47730 measured reflections	*k* = −13→13
6616 independent reflections	*l* = −18→18

### 10a-Hydroxy-9-[2-(trifluoromethyl)phenyl]-3,4,5,6,7,8a,9,10a-octahydro-1H-xanthene-1,8(2H)-dione Refinement

**Table:**

Refinement on *F*^2^	Primary atom site location: dual
Least-squares matrix: full	Secondary atom site location: difference Fourier map
*R*[*F*^2^ > 2σ(*F*^2^)] = 0.068	Hydrogen site location: mixed
*wR*(*F*^2^) = 0.150	H atoms treated by a mixture of independent and constrained refinement
*S* = 1.08	*w* = 1/[σ^2^(*F*_o_^2^) + (0.0398*P*)^2^ + 2.0212*P*] where *P* = (*F*_o_^2^ + 2*F*_c_^2^)/3
6616 reflections	(Δ/σ)_max_ < 0.001
493 parameters	Δρ_max_ = 0.49 e Å^−^^3^
0 restraints	Δρ_min_ = −0.34 e Å^−^^3^

### 10a-Hydroxy-9-[2-(trifluoromethyl)phenyl]-3,4,5,6,7,8a,9,10a-octahydro-1H-xanthene-1,8(2H)-dione Special details

**Table:**

Geometry. All esds (except the esd in the dihedral angle between two l.s. planes) are estimated using the full covariance matrix. The cell esds are taken into account individually in the estimation of esds in distances, angles and torsion angles; correlations between esds in cell parameters are only used when they are defined by crystal symmetry. An approximate (isotropic) treatment of cell esds is used for estimating esds involving l.s. planes.

### 10a-Hydroxy-9-[2-(trifluoromethyl)phenyl]-3,4,5,6,7,8a,9,10a-octahydro-1H-xanthene-1,8(2H)-dione Fractional atomic coordinates and isotropic or equivalent isotropic displacement parameters (Å^2^)

**Table:**

	*x*	*y*	*z*	*U*_iso_*/*U*_eq_	
O1	0.04685 (19)	0.68197 (16)	0.53497 (12)	0.0415 (5)	
O2	−0.0425 (2)	0.87204 (19)	0.56390 (14)	0.0438 (5)	
H2	−0.079 (3)	0.824 (3)	0.606 (2)	0.066*	
O3	0.3864 (2)	0.7371 (2)	0.49268 (15)	0.0636 (7)	
O4	0.3004 (2)	0.6726 (2)	0.77485 (14)	0.0550 (6)	
F1	0.3711 (3)	1.0243 (3)	0.57110 (17)	0.1064 (10)	
F2	0.4615 (2)	0.9443 (3)	0.6775 (2)	0.1194 (10)	
F3	0.4163 (3)	1.1293 (3)	0.6713 (2)	0.1344 (13)	
C1	0.3690 (4)	1.0278 (4)	0.6559 (3)	0.0674 (11)	
C2	0.2420 (3)	1.0077 (3)	0.7041 (2)	0.0477 (8)	
C3	0.1932 (4)	1.0930 (3)	0.7619 (2)	0.0625 (10)	
H3	0.239538	1.159417	0.767991	0.075*	
C4	0.0780 (4)	1.0802 (3)	0.8097 (2)	0.0666 (11)	
H4	0.045018	1.138552	0.846931	0.080*	
C5	0.0118 (4)	0.9805 (3)	0.8022 (2)	0.0584 (9)	
H5	−0.065546	0.970241	0.835460	0.070*	
C6	0.0593 (3)	0.8958 (3)	0.74568 (19)	0.0465 (8)	
H6	0.013325	0.828457	0.741935	0.056*	
C7	0.1736 (3)	0.9073 (2)	0.69395 (18)	0.0379 (7)	
C8	0.2197 (3)	0.8123 (2)	0.62960 (17)	0.0357 (6)	
H8	0.314129	0.804622	0.629212	0.043*	
C9	0.1742 (3)	0.6898 (2)	0.65594 (18)	0.0360 (6)	
C10	0.2229 (3)	0.6260 (3)	0.73309 (19)	0.0417 (7)	
C11	0.1763 (4)	0.5050 (3)	0.7607 (2)	0.0552 (9)	
H11A	0.098290	0.514546	0.798925	0.066*	
H11B	0.241261	0.459728	0.793374	0.066*	
C12	0.1487 (4)	0.4354 (3)	0.6851 (2)	0.0577 (9)	
H12A	0.229107	0.413747	0.652041	0.069*	
H12B	0.109643	0.362262	0.706691	0.069*	
C13	0.0595 (3)	0.5076 (3)	0.6269 (2)	0.0473 (8)	
H13A	0.059302	0.469250	0.572560	0.057*	
H13B	−0.027678	0.509462	0.654392	0.057*	
C14	0.0984 (3)	0.6330 (2)	0.60840 (18)	0.0372 (7)	
C15	0.0550 (3)	0.8093 (2)	0.51477 (18)	0.0359 (7)	
C16	0.0307 (3)	0.8283 (3)	0.41956 (18)	0.0429 (7)	
H16A	−0.049301	0.793471	0.410035	0.052*	
H16B	0.019897	0.913485	0.404574	0.052*	
C17	0.1380 (3)	0.7741 (3)	0.3599 (2)	0.0540 (9)	
H17A	0.119369	0.793308	0.299851	0.065*	
H17B	0.143170	0.687774	0.369897	0.065*	
C18	0.2669 (3)	0.8221 (3)	0.3760 (2)	0.0583 (9)	
H18A	0.335698	0.783960	0.339382	0.070*	
H18B	0.264485	0.907600	0.361675	0.070*	
C19	0.2922 (3)	0.7966 (3)	0.4700 (2)	0.0451 (7)	
C20	0.1882 (3)	0.8468 (2)	0.53492 (17)	0.0350 (6)	
H20	0.186337	0.934216	0.526943	0.042*	
O5	0.52022 (19)	0.45581 (17)	0.77075 (11)	0.0395 (5)	
O6	0.4547 (2)	0.56409 (19)	0.89156 (13)	0.0429 (5)	
H6A	0.401 (3)	0.592 (3)	0.855 (2)	0.064*	
O7	0.8591 (2)	0.3713 (2)	0.82021 (16)	0.0597 (6)	
O8	0.8265 (2)	0.7386 (2)	0.68871 (13)	0.0546 (6)	
F4	0.9888 (2)	0.6603 (3)	0.90155 (18)	0.1099 (10)	
F5	0.8719 (2)	0.5522 (2)	0.98249 (19)	0.0968 (9)	
F6	0.9442 (3)	0.7017 (3)	1.03118 (19)	0.1174 (11)	
C21	0.8932 (3)	0.6659 (3)	0.9643 (2)	0.0536 (9)	
C22	0.7793 (3)	0.7418 (3)	0.93647 (18)	0.0394 (7)	
C23	0.7544 (3)	0.8500 (3)	0.9753 (2)	0.0483 (8)	
H23	0.806566	0.869800	1.017658	0.058*	
C24	0.6539 (4)	0.9279 (3)	0.9519 (2)	0.0570 (9)	
H24	0.637967	1.000232	0.978143	0.068*	
C25	0.5769 (3)	0.8985 (3)	0.8894 (2)	0.0537 (9)	
H25	0.508855	0.951256	0.872990	0.064*	
C26	0.5999 (3)	0.7914 (3)	0.85095 (19)	0.0436 (7)	
H26	0.546408	0.772878	0.808982	0.052*	
C27	0.7011 (3)	0.7096 (2)	0.87305 (17)	0.0354 (6)	
C28	0.7244 (3)	0.5918 (2)	0.82750 (16)	0.0336 (6)	
H28	0.818334	0.575745	0.820355	0.040*	
C29	0.6773 (3)	0.5996 (2)	0.73701 (17)	0.0333 (6)	
C30	0.7382 (3)	0.6793 (3)	0.67074 (18)	0.0398 (7)	
C31	0.6901 (3)	0.6886 (3)	0.58137 (19)	0.0535 (9)	
H31A	0.758336	0.714790	0.539497	0.064*	
H31B	0.618061	0.748161	0.579322	0.064*	
C32	0.6475 (3)	0.5703 (3)	0.55668 (19)	0.0523 (9)	
H32A	0.721407	0.512742	0.552333	0.063*	
H32B	0.611718	0.580587	0.500382	0.063*	
C33	0.5470 (3)	0.5239 (3)	0.62410 (17)	0.0430 (7)	
H33A	0.465701	0.570467	0.617923	0.052*	
H33B	0.533717	0.441438	0.614226	0.052*	
C34	0.5866 (3)	0.5310 (3)	0.71430 (17)	0.0347 (6)	
C35	0.5311 (3)	0.4650 (3)	0.86283 (16)	0.0356 (7)	
C36	0.4825 (3)	0.3502 (3)	0.9050 (2)	0.0481 (8)	
H36A	0.396614	0.340900	0.887314	0.058*	
H36B	0.476211	0.354804	0.967630	0.058*	
C37	0.5696 (4)	0.2417 (3)	0.8810 (2)	0.0615 (10)	
H37A	0.569984	0.232216	0.819105	0.074*	
H37B	0.536897	0.170581	0.911653	0.074*	
C38	0.7067 (4)	0.2559 (3)	0.9048 (3)	0.0671 (11)	
H38A	0.707532	0.256271	0.967515	0.081*	
H38B	0.762475	0.188339	0.885607	0.081*	
C39	0.7573 (3)	0.3695 (3)	0.8637 (2)	0.0451 (8)	
C40	0.6712 (3)	0.4828 (2)	0.87970 (17)	0.0349 (6)	
H40	0.672393	0.497469	0.941421	0.042*	

### 10a-Hydroxy-9-[2-(trifluoromethyl)phenyl]-3,4,5,6,7,8a,9,10a-octahydro-1H-xanthene-1,8(2H)-dione Atomic displacement parameters (Å^2^)

**Table:**

	*U*^11^	*U*^22^	*U*^33^	*U*^12^	*U*^13^	*U*^23^
O1	0.0531 (13)	0.0306 (11)	0.0429 (11)	−0.0079 (9)	−0.0173 (9)	0.0036 (9)
O2	0.0420 (12)	0.0407 (12)	0.0470 (12)	−0.0020 (10)	0.0029 (10)	0.0024 (10)
O3	0.0480 (14)	0.0805 (18)	0.0593 (15)	0.0146 (13)	0.0011 (12)	−0.0070 (13)
O4	0.0642 (15)	0.0517 (14)	0.0507 (13)	0.0026 (11)	−0.0248 (12)	0.0013 (11)
F1	0.0952 (19)	0.155 (3)	0.0752 (17)	−0.0707 (18)	0.0063 (14)	−0.0001 (16)
F2	0.0553 (15)	0.141 (3)	0.159 (3)	−0.0068 (16)	−0.0117 (16)	0.022 (2)
F3	0.118 (2)	0.114 (2)	0.182 (3)	−0.080 (2)	0.019 (2)	−0.051 (2)
C1	0.064 (3)	0.066 (3)	0.077 (3)	−0.030 (2)	−0.021 (2)	0.000 (2)
C2	0.053 (2)	0.0436 (19)	0.0494 (18)	−0.0087 (15)	−0.0183 (15)	−0.0015 (15)
C3	0.083 (3)	0.042 (2)	0.068 (2)	−0.0047 (19)	−0.029 (2)	−0.0146 (18)
C4	0.089 (3)	0.060 (2)	0.054 (2)	0.010 (2)	−0.018 (2)	−0.0252 (18)
C5	0.064 (2)	0.064 (2)	0.0469 (19)	0.0040 (19)	−0.0018 (16)	−0.0138 (17)
C6	0.052 (2)	0.0456 (19)	0.0421 (17)	−0.0054 (15)	−0.0042 (15)	−0.0050 (14)
C7	0.0436 (17)	0.0342 (16)	0.0370 (15)	−0.0015 (13)	−0.0133 (13)	−0.0001 (12)
C8	0.0315 (15)	0.0374 (16)	0.0388 (15)	−0.0039 (12)	−0.0062 (12)	−0.0007 (13)
C9	0.0371 (16)	0.0326 (16)	0.0377 (15)	0.0003 (12)	−0.0033 (12)	−0.0007 (12)
C10	0.0447 (18)	0.0415 (18)	0.0380 (16)	0.0049 (14)	−0.0057 (14)	0.0000 (14)
C11	0.063 (2)	0.051 (2)	0.0500 (19)	−0.0041 (17)	−0.0123 (16)	0.0155 (16)
C12	0.067 (2)	0.0393 (19)	0.065 (2)	−0.0029 (17)	−0.0107 (18)	0.0100 (16)
C13	0.060 (2)	0.0325 (17)	0.0505 (19)	−0.0058 (15)	−0.0114 (15)	0.0023 (14)
C14	0.0384 (16)	0.0329 (16)	0.0398 (16)	−0.0018 (13)	−0.0041 (13)	0.0017 (13)
C15	0.0403 (16)	0.0286 (15)	0.0384 (15)	−0.0027 (13)	−0.0047 (13)	0.0031 (12)
C16	0.0499 (19)	0.0389 (17)	0.0407 (17)	−0.0037 (14)	−0.0111 (14)	0.0022 (13)
C17	0.069 (2)	0.055 (2)	0.0382 (17)	0.0025 (17)	−0.0087 (16)	−0.0047 (15)
C18	0.058 (2)	0.073 (2)	0.0411 (18)	−0.0002 (18)	0.0060 (16)	−0.0005 (17)
C19	0.0421 (19)	0.0486 (19)	0.0442 (18)	−0.0063 (15)	0.0018 (14)	−0.0025 (14)
C20	0.0381 (16)	0.0319 (15)	0.0353 (15)	−0.0034 (12)	−0.0060 (12)	−0.0001 (12)
O5	0.0469 (12)	0.0437 (12)	0.0294 (10)	−0.0101 (9)	−0.0047 (9)	−0.0023 (9)
O6	0.0438 (12)	0.0473 (13)	0.0371 (11)	0.0093 (10)	−0.0078 (9)	−0.0070 (9)
O7	0.0517 (15)	0.0551 (15)	0.0696 (16)	0.0111 (11)	0.0043 (13)	−0.0107 (12)
O8	0.0544 (14)	0.0683 (16)	0.0431 (12)	−0.0248 (12)	−0.0008 (10)	0.0002 (11)
F4	0.0603 (15)	0.158 (3)	0.104 (2)	0.0293 (16)	0.0009 (14)	−0.0002 (18)
F5	0.0913 (17)	0.0607 (15)	0.145 (2)	0.0035 (12)	−0.0719 (17)	0.0120 (14)
F6	0.123 (2)	0.122 (2)	0.120 (2)	0.0422 (18)	−0.0879 (18)	−0.0622 (18)
C21	0.053 (2)	0.061 (2)	0.0498 (19)	−0.0016 (17)	−0.0139 (17)	−0.0173 (17)
C22	0.0419 (17)	0.0417 (17)	0.0350 (15)	−0.0018 (14)	−0.0047 (13)	−0.0033 (13)
C23	0.062 (2)	0.0445 (19)	0.0405 (17)	−0.0080 (16)	−0.0055 (15)	−0.0099 (15)
C24	0.079 (3)	0.0370 (19)	0.054 (2)	0.0008 (18)	0.0000 (18)	−0.0100 (16)
C25	0.064 (2)	0.0389 (19)	0.056 (2)	0.0108 (16)	−0.0069 (17)	0.0004 (16)
C26	0.0496 (19)	0.0393 (18)	0.0417 (17)	0.0004 (14)	−0.0083 (14)	−0.0001 (14)
C27	0.0406 (16)	0.0364 (16)	0.0285 (14)	−0.0014 (13)	−0.0014 (12)	0.0008 (12)
C28	0.0328 (15)	0.0368 (16)	0.0314 (14)	0.0035 (12)	−0.0069 (12)	−0.0033 (12)
C29	0.0338 (15)	0.0361 (16)	0.0295 (14)	−0.0004 (12)	−0.0019 (11)	−0.0025 (12)
C30	0.0370 (17)	0.0465 (18)	0.0353 (16)	−0.0015 (14)	−0.0008 (13)	−0.0021 (13)
C31	0.053 (2)	0.071 (2)	0.0368 (17)	−0.0166 (17)	−0.0049 (15)	0.0141 (16)
C32	0.0465 (19)	0.082 (3)	0.0292 (15)	−0.0087 (17)	−0.0017 (13)	−0.0043 (16)
C33	0.0455 (18)	0.0526 (19)	0.0323 (15)	−0.0075 (15)	−0.0069 (13)	−0.0045 (14)
C34	0.0352 (15)	0.0396 (16)	0.0286 (14)	−0.0014 (13)	−0.0001 (12)	−0.0018 (12)
C35	0.0435 (17)	0.0385 (16)	0.0245 (13)	−0.0017 (13)	−0.0026 (12)	−0.0020 (12)
C36	0.057 (2)	0.0471 (19)	0.0390 (17)	−0.0092 (16)	0.0020 (15)	0.0031 (14)
C37	0.081 (3)	0.0358 (19)	0.065 (2)	−0.0085 (18)	0.0079 (19)	0.0013 (16)
C38	0.076 (3)	0.039 (2)	0.081 (3)	0.0120 (18)	0.005 (2)	0.0083 (18)
C39	0.053 (2)	0.0399 (18)	0.0426 (17)	0.0055 (15)	−0.0090 (15)	−0.0030 (14)
C40	0.0395 (16)	0.0356 (16)	0.0291 (14)	0.0040 (13)	−0.0049 (12)	−0.0017 (12)

### 10a-Hydroxy-9-[2-(trifluoromethyl)phenyl]-3,4,5,6,7,8a,9,10a-octahydro-1H-xanthene-1,8(2H)-dione Geometric parameters (Å, º)

**Table:**

O1—C14	1.360 (3)	O5—C34	1.356 (3)
O1—C15	1.449 (3)	O5—C35	1.448 (3)
O2—C15	1.397 (3)	O6—C35	1.397 (3)
O2—H2	0.89 (4)	O6—H6A	0.86 (4)
O3—C19	1.208 (4)	O7—C39	1.209 (4)
O4—C10	1.236 (4)	O8—C30	1.232 (3)
F1—C1	1.313 (4)	F4—C21	1.335 (4)
F2—C1	1.340 (5)	F5—C21	1.319 (4)
F3—C1	1.316 (4)	F6—C21	1.297 (4)
C1—C2	1.489 (5)	C21—C22	1.485 (4)
C2—C3	1.395 (5)	C22—C23	1.390 (4)
C2—C7	1.399 (4)	C22—C27	1.403 (4)
C3—C4	1.369 (5)	C23—C24	1.370 (5)
C3—H3	0.9300	C23—H23	0.9300
C4—C5	1.372 (5)	C24—C25	1.375 (5)
C4—H4	0.9300	C24—H24	0.9300
C5—C6	1.374 (4)	C25—C26	1.373 (4)
C5—H5	0.9300	C25—H25	0.9300
C6—C7	1.389 (4)	C26—C27	1.395 (4)
C6—H6	0.9300	C26—H26	0.9300
C7—C8	1.533 (4)	C27—C28	1.534 (4)
C8—C9	1.505 (4)	C28—C29	1.513 (4)
C8—C20	1.543 (4)	C28—C40	1.533 (4)
C8—H8	0.9800	C28—H28	0.9800
C9—C14	1.340 (4)	C29—C34	1.342 (4)
C9—C10	1.457 (4)	C29—C30	1.452 (4)
C10—C11	1.499 (4)	C30—C31	1.501 (4)
C11—C12	1.504 (5)	C31—C32	1.511 (5)
C11—H11A	0.9700	C31—H31A	0.9700
C11—H11B	0.9700	C31—H31B	0.9700
C12—C13	1.502 (4)	C32—C33	1.511 (4)
C12—H12A	0.9700	C32—H32A	0.9700
C12—H12B	0.9700	C32—H32B	0.9700
C13—C14	1.495 (4)	C33—C34	1.493 (4)
C13—H13A	0.9700	C33—H33A	0.9700
C13—H13B	0.9700	C33—H33B	0.9700
C15—C16	1.511 (4)	C35—C36	1.507 (4)
C15—C20	1.537 (4)	C35—C40	1.531 (4)
C16—C17	1.511 (4)	C36—C37	1.514 (5)
C16—H16A	0.9700	C36—H36A	0.9700
C16—H16B	0.9700	C36—H36B	0.9700
C17—C18	1.525 (5)	C37—C38	1.522 (5)
C17—H17A	0.9700	C37—H37A	0.9700
C17—H17B	0.9700	C37—H37B	0.9700
C18—C19	1.501 (4)	C38—C39	1.497 (5)
C18—H18A	0.9700	C38—H38A	0.9700
C18—H18B	0.9700	C38—H38B	0.9700
C19—C20	1.521 (4)	C39—C40	1.526 (4)
C20—H20	0.9800	C40—H40	0.9800
			
C14—O1—C15	118.1 (2)	C34—O5—C35	117.9 (2)
C15—O2—H2	111 (2)	C35—O6—H6A	113 (2)
F1—C1—F3	106.8 (3)	F6—C21—F5	106.4 (3)
F1—C1—F2	103.2 (4)	F6—C21—F4	105.5 (3)
F3—C1—F2	104.1 (3)	F5—C21—F4	102.5 (3)
F1—C1—C2	115.3 (3)	F6—C21—C22	114.6 (3)
F3—C1—C2	113.7 (4)	F5—C21—C22	114.6 (3)
F2—C1—C2	112.6 (3)	F4—C21—C22	112.1 (3)
C3—C2—C7	120.4 (3)	C23—C22—C27	120.6 (3)
C3—C2—C1	117.2 (3)	C23—C22—C21	116.4 (3)
C7—C2—C1	122.4 (3)	C27—C22—C21	123.0 (3)
C4—C3—C2	120.8 (3)	C24—C23—C22	120.7 (3)
C4—C3—H3	119.6	C24—C23—H23	119.6
C2—C3—H3	119.6	C22—C23—H23	119.6
C3—C4—C5	119.4 (3)	C23—C24—C25	119.5 (3)
C3—C4—H4	120.3	C23—C24—H24	120.2
C5—C4—H4	120.3	C25—C24—H24	120.2
C4—C5—C6	120.2 (4)	C26—C25—C24	120.3 (3)
C4—C5—H5	119.9	C26—C25—H25	119.9
C6—C5—H5	119.9	C24—C25—H25	119.9
C5—C6—C7	122.2 (3)	C25—C26—C27	121.9 (3)
C5—C6—H6	118.9	C25—C26—H26	119.0
C7—C6—H6	118.9	C27—C26—H26	119.0
C6—C7—C2	116.9 (3)	C26—C27—C22	116.9 (3)
C6—C7—C8	120.1 (3)	C26—C27—C28	120.2 (2)
C2—C7—C8	123.0 (3)	C22—C27—C28	122.9 (2)
C9—C8—C7	114.0 (2)	C29—C28—C40	109.5 (2)
C9—C8—C20	109.2 (2)	C29—C28—C27	113.3 (2)
C7—C8—C20	114.2 (2)	C40—C28—C27	114.6 (2)
C9—C8—H8	106.3	C29—C28—H28	106.3
C7—C8—H8	106.3	C40—C28—H28	106.3
C20—C8—H8	106.3	C27—C28—H28	106.3
C14—C9—C10	118.2 (3)	C34—C29—C30	118.6 (2)
C14—C9—C8	123.1 (3)	C34—C29—C28	122.7 (2)
C10—C9—C8	118.5 (2)	C30—C29—C28	118.6 (2)
O4—C10—C9	119.8 (3)	O8—C30—C29	120.0 (3)
O4—C10—C11	121.3 (3)	O8—C30—C31	121.4 (3)
C9—C10—C11	118.9 (3)	C29—C30—C31	118.7 (3)
C10—C11—C12	112.9 (3)	C30—C31—C32	111.7 (3)
C10—C11—H11A	109.0	C30—C31—H31A	109.3
C12—C11—H11A	109.0	C32—C31—H31A	109.3
C10—C11—H11B	109.0	C30—C31—H31B	109.3
C12—C11—H11B	109.0	C32—C31—H31B	109.3
H11A—C11—H11B	107.8	H31A—C31—H31B	107.9
C13—C12—C11	111.0 (3)	C31—C32—C33	110.2 (3)
C13—C12—H12A	109.4	C31—C32—H32A	109.6
C11—C12—H12A	109.4	C33—C32—H32A	109.6
C13—C12—H12B	109.4	C31—C32—H32B	109.6
C11—C12—H12B	109.4	C33—C32—H32B	109.6
H12A—C12—H12B	108.0	H32A—C32—H32B	108.1
C14—C13—C12	112.1 (3)	C34—C33—C32	111.7 (2)
C14—C13—H13A	109.2	C34—C33—H33A	109.3
C12—C13—H13A	109.2	C32—C33—H33A	109.3
C14—C13—H13B	109.2	C34—C33—H33B	109.3
C12—C13—H13B	109.2	C32—C33—H33B	109.3
H13A—C13—H13B	107.9	H33A—C33—H33B	107.9
C9—C14—O1	124.2 (3)	C29—C34—O5	124.1 (2)
C9—C14—C13	125.6 (3)	C29—C34—C33	125.3 (3)
O1—C14—C13	110.2 (2)	O5—C34—C33	110.7 (2)
O2—C15—O1	109.4 (2)	O6—C35—O5	109.8 (2)
O2—C15—C16	109.2 (2)	O6—C35—C36	111.1 (2)
O1—C15—C16	105.1 (2)	O5—C35—C36	104.7 (2)
O2—C15—C20	110.2 (2)	O6—C35—C40	107.9 (2)
O1—C15—C20	109.8 (2)	O5—C35—C40	109.9 (2)
C16—C15—C20	113.1 (2)	C36—C35—C40	113.4 (2)
C17—C16—C15	113.4 (2)	C35—C36—C37	112.5 (3)
C17—C16—H16A	108.9	C35—C36—H36A	109.1
C15—C16—H16A	108.9	C37—C36—H36A	109.1
C17—C16—H16B	108.9	C35—C36—H36B	109.1
C15—C16—H16B	108.9	C37—C36—H36B	109.1
H16A—C16—H16B	107.7	H36A—C36—H36B	107.8
C16—C17—C18	110.5 (3)	C36—C37—C38	110.3 (3)
C16—C17—H17A	109.5	C36—C37—H37A	109.6
C18—C17—H17A	109.5	C38—C37—H37A	109.6
C16—C17—H17B	109.5	C36—C37—H37B	109.6
C18—C17—H17B	109.5	C38—C37—H37B	109.6
H17A—C17—H17B	108.1	H37A—C37—H37B	108.1
C19—C18—C17	109.2 (3)	C39—C38—C37	111.2 (3)
C19—C18—H18A	109.8	C39—C38—H38A	109.4
C17—C18—H18A	109.8	C37—C38—H38A	109.4
C19—C18—H18B	109.8	C39—C38—H38B	109.4
C17—C18—H18B	109.8	C37—C38—H38B	109.4
H18A—C18—H18B	108.3	H38A—C38—H38B	108.0
O3—C19—C18	122.5 (3)	O7—C39—C38	122.3 (3)
O3—C19—C20	122.2 (3)	O7—C39—C40	122.2 (3)
C18—C19—C20	115.3 (3)	C38—C39—C40	115.4 (3)
C19—C20—C15	110.1 (2)	C39—C40—C35	110.9 (2)
C19—C20—C8	111.7 (2)	C39—C40—C28	111.4 (2)
C15—C20—C8	112.8 (2)	C35—C40—C28	112.7 (2)
C19—C20—H20	107.3	C39—C40—H40	107.2
C15—C20—H20	107.3	C35—C40—H40	107.2
C8—C20—H20	107.3	C28—C40—H40	107.2
			
F1—C1—C2—C3	−127.2 (4)	F6—C21—C22—C23	10.0 (5)
F3—C1—C2—C3	−3.5 (5)	F5—C21—C22—C23	133.4 (3)
F2—C1—C2—C3	114.6 (4)	F4—C21—C22—C23	−110.3 (3)
F1—C1—C2—C7	53.7 (5)	F6—C21—C22—C27	−171.5 (3)
F3—C1—C2—C7	177.5 (3)	F5—C21—C22—C27	−48.1 (4)
F2—C1—C2—C7	−64.4 (4)	F4—C21—C22—C27	68.2 (4)
C7—C2—C3—C4	0.1 (5)	C27—C22—C23—C24	−0.6 (5)
C1—C2—C3—C4	−179.0 (3)	C21—C22—C23—C24	177.9 (3)
C2—C3—C4—C5	1.7 (5)	C22—C23—C24—C25	0.1 (5)
C3—C4—C5—C6	−1.5 (5)	C23—C24—C25—C26	0.4 (5)
C4—C5—C6—C7	−0.6 (5)	C24—C25—C26—C27	−0.3 (5)
C5—C6—C7—C2	2.4 (4)	C25—C26—C27—C22	−0.2 (4)
C5—C6—C7—C8	−177.8 (3)	C25—C26—C27—C28	−179.1 (3)
C3—C2—C7—C6	−2.1 (4)	C23—C22—C27—C26	0.7 (4)
C1—C2—C7—C6	176.9 (3)	C21—C22—C27—C26	−177.8 (3)
C3—C2—C7—C8	178.1 (3)	C23—C22—C27—C28	179.5 (3)
C1—C2—C7—C8	−2.9 (5)	C21—C22—C27—C28	1.1 (4)
C6—C7—C8—C9	−27.4 (4)	C26—C27—C28—C29	26.8 (4)
C2—C7—C8—C9	152.4 (3)	C22—C27—C28—C29	−152.0 (3)
C6—C7—C8—C20	99.1 (3)	C26—C27—C28—C40	−99.8 (3)
C2—C7—C8—C20	−81.1 (3)	C22—C27—C28—C40	81.3 (3)
C7—C8—C9—C14	118.2 (3)	C40—C28—C29—C34	9.8 (4)
C20—C8—C9—C14	−10.9 (4)	C27—C28—C29—C34	−119.5 (3)
C7—C8—C9—C10	−66.8 (3)	C40—C28—C29—C30	−166.5 (2)
C20—C8—C9—C10	164.1 (2)	C27—C28—C29—C30	64.2 (3)
C14—C9—C10—O4	174.8 (3)	C34—C29—C30—O8	−176.2 (3)
C8—C9—C10—O4	−0.4 (4)	C28—C29—C30—O8	0.2 (4)
C14—C9—C10—C11	−5.8 (4)	C34—C29—C30—C31	4.6 (4)
C8—C9—C10—C11	179.0 (3)	C28—C29—C30—C31	−179.0 (3)
O4—C10—C11—C12	−147.7 (3)	O8—C30—C31—C32	146.2 (3)
C9—C10—C11—C12	32.9 (4)	C29—C30—C31—C32	−34.6 (4)
C10—C11—C12—C13	−52.5 (4)	C30—C31—C32—C33	55.5 (4)
C11—C12—C13—C14	45.6 (4)	C31—C32—C33—C34	−47.2 (4)
C10—C9—C14—O1	−179.8 (3)	C30—C29—C34—O5	−177.9 (2)
C8—C9—C14—O1	−4.8 (4)	C28—C29—C34—O5	5.8 (4)
C10—C9—C14—C13	−0.7 (4)	C30—C29—C34—C33	3.8 (4)
C8—C9—C14—C13	174.4 (3)	C28—C29—C34—C33	−172.5 (3)
C15—O1—C14—C9	−11.2 (4)	C35—O5—C34—C29	11.3 (4)
C15—O1—C14—C13	169.5 (2)	C35—O5—C34—C33	−170.3 (2)
C12—C13—C14—C9	−20.2 (4)	C32—C33—C34—C29	18.5 (4)
C12—C13—C14—O1	159.1 (3)	C32—C33—C34—O5	−159.9 (3)
C14—O1—C15—O2	−80.5 (3)	C34—O5—C35—O6	77.2 (3)
C14—O1—C15—C16	162.4 (2)	C34—O5—C35—C36	−163.4 (2)
C14—O1—C15—C20	40.5 (3)	C34—O5—C35—C40	−41.4 (3)
O2—C15—C16—C17	174.2 (2)	O6—C35—C36—C37	−175.2 (3)
O1—C15—C16—C17	−68.5 (3)	O5—C35—C36—C37	66.4 (3)
C20—C15—C16—C17	51.2 (3)	C40—C35—C36—C37	−53.5 (3)
C15—C16—C17—C18	−55.8 (4)	C35—C36—C37—C38	56.6 (4)
C16—C17—C18—C19	56.8 (4)	C36—C37—C38—C39	−55.3 (4)
C17—C18—C19—O3	120.7 (4)	C37—C38—C39—O7	−127.0 (3)
C17—C18—C19—C20	−56.9 (4)	C37—C38—C39—C40	52.5 (4)
O3—C19—C20—C15	−126.0 (3)	O7—C39—C40—C35	131.7 (3)
C18—C19—C20—C15	51.6 (3)	C38—C39—C40—C35	−47.7 (3)
O3—C19—C20—C8	0.2 (4)	O7—C39—C40—C28	5.3 (4)
C18—C19—C20—C8	177.8 (3)	C38—C39—C40—C28	−174.2 (3)
O2—C15—C20—C19	−169.5 (2)	O6—C35—C40—C39	170.8 (2)
O1—C15—C20—C19	70.0 (3)	O5—C35—C40—C39	−69.5 (3)
C16—C15—C20—C19	−47.0 (3)	C36—C35—C40—C39	47.3 (3)
O2—C15—C20—C8	65.0 (3)	O6—C35—C40—C28	−63.5 (3)
O1—C15—C20—C8	−55.6 (3)	O5—C35—C40—C28	56.2 (3)
C16—C15—C20—C8	−172.6 (2)	C36—C35—C40—C28	173.0 (2)
C9—C8—C20—C19	−84.5 (3)	C29—C28—C40—C39	85.6 (3)
C7—C8—C20—C19	146.6 (2)	C27—C28—C40—C39	−145.8 (2)
C9—C8—C20—C15	40.2 (3)	C29—C28—C40—C35	−39.8 (3)
C7—C8—C20—C15	−88.7 (3)	C27—C28—C40—C35	88.8 (3)

### 10a-Hydroxy-9-[2-(trifluoromethyl)phenyl]-3,4,5,6,7,8a,9,10a-octahydro-1H-xanthene-1,8(2H)-dione Hydrogen-bond geometry (Å, º)

**Table:**

*D*—H···*A*	*D*—H	H···*A*	*D*···*A*	*D*—H···*A*
O2—H2···O8^i^	0.89 (4)	1.82 (4)	2.704 (3)	172 (3)
C11—H11*A*···F4^i^	0.97	2.51	3.307 (5)	140
C16—H16*B*···O2^ii^	0.97	2.52	3.403 (4)	152
C20—H20···F1	0.98	2.40	2.960 (4)	116
O6—H6*A*···O4	0.86 (4)	1.83 (4)	2.684 (3)	171 (3)
C36—H36*B*···O6^iii^	0.97	2.59	3.473 (4)	152
C40—H40···F5	0.98	2.35	2.901 (4)	115

Symmetry codes: (i) *x*−1, *y*, *z*; (ii) −*x*, −*y*+2, −*z*+1; (iii) −*x*+1, −*y*+1, −*z*+2.
